# Oat Meal

**Published:** 1891-06

**Authors:** 


### Oat Meal.

A strong, hearty, baggage-man who could, with apparent ease, elevate a modern Saratoga trunk, was recently the object of the most intense admiration of a boy of nine, whose greatest ambition is to be strong and who would rather be John Sullivan than President Harrison. Thinking it a good opportunity for an object lesson in eating (as this boy was not what he should be in this line), we asked this Hercules, in the presence of the boy, what he ate to make him so strong. Oatmeal, was the reply; a good, big dish of it every morning for breakfast; there is nothing better, he added, and being cheap, it is within the reach of all, even the poorest. The practical knowledge of this strong baggageman is corroborated by science, and we would that well-cooked oat meal was even more freely used than it is.—[*Annals of Hygiene.*

